# Parents’ Awareness and Knowledge of G6PD Deficiency in the Eastern Province Saudi Arabia: A Cross-Sectional Study

**DOI:** 10.7759/cureus.50845

**Published:** 2023-12-20

**Authors:** Hwazen Shash, Mohammed Alomari, Ammar Alsaif, Ali Abualrahi, Mohammad AlQassab, Ammar Alfaraj, Ali Alkhadhabah, Abdullah Alhajji

**Affiliations:** 1 College of Medicine, Imam Abdulrahman Bin Faisal University, Dammam, SAU; 2 Department of Pediatrics, King Fahd Hospital of the University, Khobar, SAU

**Keywords:** hematology, g6pd, pediatrics, glucose-6-phosphate dehydrogenase (g6pd) deficiency, anemia

## Abstract

Background

Glucose-6-phosphate dehydrogenase deficiency, known as G6PD deficiency, is a common hematological disease in the Eastern Province. The presence of Glucose-6-phosphate dehydrogenase enzyme in the erythrocyte is crucial, as it aids in the protection of RBCs by preventing cellular damage. It was found that more than 400 million people in the world lack this enzyme, making it the most common enzyme deficiency worldwide. Because of the high prevalence of the disease in the world and the paucity of research in Saudi Arabia about G6PD deficiency, the idea of examining and assessing the awareness and knowledge of parents who have children affected with G6PD emerged.

Objective

This study aimed to evaluate parental knowledge regarding G6PD deficiency, identify the common and spread misconceptions regarding the disease, provide general insight for physicians about parental knowledge, and propose strategies to educate parents about G6PD deficiency.

Methods

This cross-sectional study was conducted from September 2022 to May 2023 on 459 individuals from the Eastern Province, Saudi Arabia. Using a questionnaire, parents with variable education levels and incomes participated. The questionnaire was available in both Arabic and English. The study aimed to gather comprehensive data regarding parental awareness and knowledge of G6PD deficiency, it focused on evaluating levels of awareness, knowledge, and misinformation of the participants. Data were analyzed by using Statistical Package for the Social Sciences, ver 22 (IBM Corp., Armonk, NY). The chi-square test was applied to check any association between demographics and level of knowledge (Good and Poor). Multiple logistic regression analysis was performed on significant demographic variables and an odds ratio with a 95% confidence interval was calculated. P < 0.05 was considered statistically significant.

Results

The study included a total of 459 participants, 309 (67.3%) were females and 150 (32.7%) were males. Regarding the demographics, the majority of the participants were <40 years old (61.9%), Saudi (99.3%), married (98.5%), having bachelor’s/diploma level or above (Master/PhD) (76.9%), having inherited disorder (44.2%), and having a family history of chronic diseases (82.6%). Furthermore, regarding the knowledge of the disease, the majority of the participants who recognized the disease (82.1%) were familiar with the term fava bean anemia rather than it being called G6PD deficiency anemia. On the other hand, 73.2% of the participants were unable to recognize the disease and had never heard of G6PD deficiency anemia. In addition, the majority of participants had deficient information regarding medication triggers (61.4%), whether the gender of a person is linked to G6PD (77.5%), and whether both parents must be carriers to have a child with G6PD deficiency anemia (50.9%). Female respondents proved to be more knowledgeable regarding this topic as 57.9% of them showed good knowledge as compared to (44.7%) of the male subjects.

Conclusion

There are discrepancies in the level of awareness among research participants. Our result indicates the need for educational interventions regarding the nomenclature, medication triggers, inheritance mode and its relation to gender, and the symptoms of the disease and its severity. Therefore, it is advised to spread awareness in the eastern province through brochures, medical campaigns, and by healthcare professionals in different medical organizations.

## Introduction

Glucose-6-phosphate dehydrogenase deficiency, known as G6PD deficiency, is the most common metabolic red blood cell defect, which is inherited in an X-linked fashion [1]. It affects more than 400 million people worldwide [1]. It affects males predominantly due to the mode of inheritance, but females can be affected as well [1]. The G6PD enzyme is an essential part of the pentose phosphate pathway, which aids red blood cells' survival, as it prevents cellular damage [1-6]. When the G6PD enzyme is deficient, oxidized nicotinamide adenine dinucleotide phosphate (NADP) does not get reduced to NADPH. The importance of NADPH is evident, as it helps in the conversion process of toxic oxygen radicals into non-harmful H2O [5]. When this biochemical process is disrupted because of the reduction of the G6PD enzyme in the cells, accumulation of free oxygen species is triggered by the administration of specific medications, ingestion of some types of foods like fava beans, exposure to certain chemicals, or infections [7]. Generally, the clinical manifestations of such defects are variable in presentation and severity, but hemolysis is the notable feature [1].

G6PD deficiency is common in Asia, Africa, the Middle East, and the Mediterranean, which are considered subtropical and tropical areas [8]. In Saudi Arabia, the prevalence is very high reaching 8.4% of afflicted males [8]. Moreover, one in every 4.3 newborn babies in the Eastern Province is born with G6PD deficiency. A G6PD deficiency screening program was conducted in 2020 with a total of 48,889 patients, comprising 27,634 males and 21,255 females, with a mean age of 1.93 + 3.98 years [9]. In that screening program, 25% of the total population turned out to be G6PD deficient, affecting 33.8% of the male participants and 13.2% of the female participants, showing the higher prevalence of this disease among males [9].

Despite the high prevalence of G6PD deficiency in Saudi Arabia, more importantly in Eastern Province, the reported studies were few regarding the community awareness of G6PD deficiency. Due to the limited research available and the considerable impact of G6PD deficiency on patients' well-being, conducting a study to evaluate the level of awareness and understanding regarding this condition within the region became crucial. This study aims to assess parental awareness of G6PD deficiency, including understanding its inheritance, biochemical mechanisms, triggers, and clinical manifestations. Additionally, it seeks to compare awareness levels among parents across various demographic backgrounds to identify potential variations in knowledge and misconceptions related to G6PD deficiency.

## Materials and methods

Study overview

The study was designed as a cross-sectional study, selected to encompass multiple variables such as sex, diverse age groups, educational levels, marital status, the presence of G6PD deficiency in participants, and whether their children have G6PD deficiency. Its primary objective was to assess the level of parents’ knowledge and awareness regarding the different names of G6PD deficiency, mode of inheritance, triggers, clinical presentation, and potential complications that may occur secondary to the hemolysis that may be induced by the deficiency of this crucial enzyme.

Ethical considerations

This study was reviewed by the Institutional Review Board Committee at Imam Abdulrahman bin Faisal University (IRB-UGS-2022-01-420). The consent of each participant was obtained at the start of the questionnaire, and they were informed about the study having true and false information. The participants choose to withdraw from the study at any point in time they wish. The information and data collected will only be used for this study and will be kept with the highest confidentiality.

Study population

Conducted within the Eastern Province of Saudi Arabia, the study encompassed all geographical areas within this region while excluding those that were not. The study encompassed married or previously married individuals, both genders, irrespective of their G6PD status. It included people of any age and included individuals without children in its scope.

Study procedure

The research methodology encompassed a structured approach to ensure a comprehensive investigation. Initially, a questionnaire was distributed between the first of November 2022 to the 31st of December 2022 across diverse social media platforms (i.e. Twitter, WhatsApp, Instagram) to collect responses from participants (see Appendices for the questionnaire). Following the data collection phase, a systematic analysis was conducted using the Statistical Package for the Social Sciences (SPSS) version 22.0 (IBM Corp., Armonk, NY). This software facilitated precise data organization and statistical examination, allowing for the identification of significant trends, correlations, and patterns within the collected information. Furthermore, the research incorporated an extensive review and collection of relevant literature, which constituted an exploration of different scientific databases.

Instrument

The data were collected by a pre-validated questionnaire designed by Hamali HA in his paper study [8]. It contained two sections. The first section was general information about the participant. The second section was about the general knowledge of the participants about the disease, and their awareness of its symptoms, triggers, and misconceptions.

Data collection method

It was an online-based questionnaire in English and Arabic. An assessment of awareness, knowledge, and false information was done. The total number of questions was 27. The first section consisted of general information about the participant, and it had seven questions. It showed the level of education, income, ethnicity, marital status, and the use of medication if the participant had any relatives with inherited disorders. The second part of the questionnaire contained 20 questions. It asked questions about the general knowledge of the disease, mode of inheritance, neonatal screenings, some triggers, some symptoms, some complications, and the source of information about the disease. It was used without modifications and contained true and false information in yes, no, and I-don’t-know-based questions.

True information was coded as one and false and I don’t know was coded as zero. Twenty items were collected in two main categories with a total score of 19. The mean of each category and the overall mean were calculated. The first category was about the general knowledge and triggers of G6PD (11 items) with a calculated mean score of 5.5, and the second domain was about symptoms and misconceptions of G6PD (eight items) with a calculated mean score of four. The total of the items was 20 with an overall calculated mean and maximum scores of 9.5 and 19 points, respectively. Participants' mean scores of greater than the overall mean value were assumed to be good knowledge. Less than or equal to the overall mean score was assumed to be poor knowledge.

Sample size and technique

Sample Size

The sample size was estimated according to the following equation: Sample size = {Z2 x P(1-P)} / d2 [10].

where Z = reliability coefficient (Z = 1.96 at 95% confidence interval); P = is the expected prevalence; d = precision (corresponding to effect size)

The study on knowledge of and misconceptions about sickle cell anemia and glucose-6-phosphate dehydrogenase deficiency among adult sickle cell anemia patients in the Al-Qatif area (eastern KSA), where our study is based, reported that the prevalence of good knowledge was 0.437, with absolute precision of 8% (i.e., a range of prevalence of 42-58%) [11]. The sample size was calculated as 192.88.

Technique

A voluntary sampling technique was used in this study.

Statistical analysis

Data were analyzed by using Statistical Package for the Social Sciences (SPSS) version 22.0 software (IBM Corp.). All variables were presented as frequencies and percentages. The chi-square test was applied to check any association between demographics and level of knowledge (Good and Poor). Multiple logistic regression analysis was performed on significant demographic variables and an odds ratio with a 95% confidence interval was calculated. P < 0.05 was considered statistically significant.

## Results

A total of 459 parents participated in the study. Table 1 shows that more than half the participants 309 (67.3%) were females while only 150 (32.7%) responses came from males. Most of the participants (284; 61.9%) were aged > 40 years with the remaining 175 (38.1%) belonging to the 20-40 years age group. The educational level of 353 (76.9%) participants in this study was bachelor's/diploma level or above (Master's or PhD). Almost all the participants (452; 98.5%) were married. The family income of 185 (40.3%) participants was > 15000 SAR, and 456 (99.3%) responses came from Saudi individuals. Family history of chronic disease was reported in 379 (82.6%) participants, and out of the total, 203 (44.2%) respondents had an inherited disorder. The inherited disorders of the participants were noted as follows: sickle cell disease in 70 (34.5%) respondents, anemia in 54 (26.6%), 37 (18.2%) had G6PD, and 33 (16.3%) had thalassemia. Medication use was positive in 114 (24.8%) participants.

**Table 1:** Sociodemographic characteristics of the participants (n = 459) SCD: sickle cell disease

-	-	Frequency	Percent
Sex	Male	150	32.7
	Female	309	67.3
Age	20 - 40	175	38.1
	> 40	284	61.9
Education	Pre-secondary	23	5
	High school	83	18.1
	Bachelor's / Diploma / Masters or PhD	353	76.9
Marital Status	Married	452	98.5
	Divorced	7	1.5
Family monthly income	< 5000	44	9.6
	5000 - 10000	102	22.2
	10000 - 15000	128	27.9
	> 15000	185	40.3
Nationality	Saudi	456	99.3
	Non-Saudi	3	0.7
Do you have any inherited disorders?	Yes	203	44.2
	No	256	55.8
Type of disorder (If Yes) n = 203	SCD	70	34.5
	Anemia	54	26.6
	G6pd	37	18.2
	Thalassemia	33	16.3
	Others	9	4.4
Medication	Yes	114	24.8
	No	345	75.2
Family history of diseases	Yes	379	82.6
	No	80	17.4

Table 2 depicts the general knowledge of the participants towards G6PD anemia. The majority of respondents (336; 73.2%) had never heard about G6PD anemia, but 377 (82.1%) participants were familiar with fava bean anemia. A large number of participants (369; 80.4%) believed that G6PD is an inherited disorder with 234 (50.9%) participants, stating that both parents must be carriers to have a child with G6PD deficiency anemia. Of the study population, 356 (77.5%) of the participants were unfamiliar with whether the gender of a person is linked with G6PD deficiency anemia. Almost all participants (415; 90.4%) believed that neonatal diagnosis of G6PD deficiency anemia is beneficial to a child's health. Out of the total, 346 (75.4%) recognized that fava beans can trigger G6PD deficiency anemia while half the respondents (234; 50.9%) did not know the role of medications as a trigger. This reflects fluctuating levels of awareness and understanding of G6PD anemia among the surveyed participants. While certain aspects, such as the nature of the disease, were recognized, there were gaps in knowledge when it came to the inheritance pattern, gender, and triggers of G6PD anemia.

**Table 2:** General awareness of the participants toward G6PD (n = 459) G6PD: glucose-6-phosphate dehydrogenase

-	Yes	No	Do Not Know
Have you ever heard of G6PD deficiency anemia?	123 (26.8%)	336 (73.2%)	N/A
Have you heard about fava bean anemia?	377 (82.1%)	82 (17.9%)	N/A
Is G6PD deficiency a type of anemia?	324 (70.6%)	35 (7.6%)	100 (21.8%)
Do you believe that for a child to be affected with G6PD deficiency anemia, both parents must be carriers?	234 (50.9%)	149 (32.5%)	76 (16.6%)
Do you think G6PD deficiency is an inherited disorder?	369 (80.4%)	25 (5.4%)	65 (14.2%)
Do you know if you have G6PD deficiency anemia?	237 (51.6%)	222 (48.4%)	N/A
Do you have a family history of G6PD deficiency anemia?	280 (61%)	106 (23.1%)	73 (15.9%)
Is the gender of the baby linked to G6PD deficiency anemia?	103 (22.4%)	195 (42.5%)	161 (35.1%)
Do you presume that the neonatal diagnosis of G6PD deficiency anemia is beneficial to your child's care?	415 (90.4%)	11 (2.4%)	33 (7.2%)
Can Fava beans trigger an attack of G6PD deficiency anemia?	346 (75.4%)	20 (4.4%)	93 (20.3%)
Can certain medications trigger an attack of G6PD deficiency anemia?	177 (38.6%)	48 (10.5%)	234 (50.9%)

It is apparent from Table 3 that more than 50% of the study population was deficient in knowledge regarding some of the symptoms of a G6PD anemia attack such as nausea, vomiting, anorexia, diarrhea, and shortness of breath. However, other symptoms were fairly recognized by the participants; these include pallor, which was seen in 338 (73.6%) participants, dizziness in 262 (57.1%) participants, and jaundice in 378 (69.3%) participants. Half of the study population was unclear on the role of laboratory tests for G6PD deficiency anemia in premarital testing. Many of the participants underestimated G6PD with 349 (76%) participants not believing that G6PD deficiency anemia can lead to death or handicap. Moreover, 399 (86.9%) participants were unaware of the effect of splenectomy on G6PD patients’ health. These data reveal insufficient knowledge of the symptoms of G6PD, premarital testing, and the severity of the potential outcomes among the surveyed participants.

**Table 3:** Knowledge of the participants of the symptoms of G6PD (n = 459) G6PD: glucose-6-phosphate dehydrogenase

-	Yes	No	Don’t Know
Are nausea, vomiting, anorexia, and diarrhea symptoms of a G6PD deficiency anemia attack?	121 (26.4%)	97 (21.1%)	241 (52.5%)
Is pallor one of the symptoms of a G6PD deficiency anemia attack?	326 (71%)	18 (3.9%)	115 (25.1%)
Is dizziness one of the symptoms of a G6PD deficiency anemia attack?	262 (57.1%)	36 (7.8%)	161 (35.1%)
Is jaundice one of the symptoms of a G6PD deficiency anemia attack?	318 (69.3%)	23 (5%)	118 (25.7%)
Is shortness of breath one of the symptoms of a G6PD deficiency anemia attack?	185 (40.3%)	69 (15%)	205 (44.7%)
Do you think that a laboratory test for G6PD deficiency anemia is included in premarital testing?	215 (46.8%)	115 (25.1%)	129 (28.1%)
Can splenectomy improve the health of patients with G6PD deficiency anemia?	60 (13.1%)	78 (17%)	321 (69.9%)
Do you believe that G6PD deficiency anemia can lead to death or handicap?	110 (24%)	149 (32.5%)	200 (43.6%)

**Figure 1:**
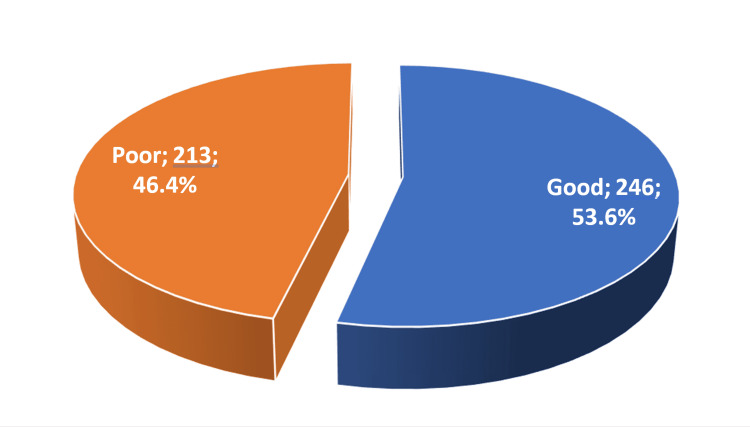
Level of knowledge toward G6PD (n = 459) G6PD: glucose-6-phosphate dehydrogenase

Figure 1 illustrates the participants’ level of knowledge about G6PD deficiency anemia with half of the participants (246; 53.6%) having good knowledge.

Table 4 shows the association between knowledge and sociodemographic characteristics. Good knowledge is assumed when the participants’ mean score is greater than the overall mean while being less than or equal to the overall mean is assumed to be poor knowledge. A significant distinction in knowledge was observed when it came to gender (p-value = 0.008) with females demonstrating a higher "Good" knowledge proportion (57.9%) compared to males (44.7%). However, no substantial difference was found between distinct age groups (p-value = 0.42), indicating similar proportions of "Good" and "Poor" knowledge in individuals over 40 and within the 20-40 age range. Marital status (p-value = 0.56) and educational levels (p-value = 0.174) showed no considerable variance in knowledge levels. Similarly, family monthly income (p-value = 0.788) exhibited consistent knowledge proportions across different income brackets. Notably, individuals with an inherited disorder displayed a statistically significant difference in knowledge compared to those without (p-value = 0.021). This was reflected in a higher percentage of individuals with an inherited disorder (59.6%) demonstrating "Good" knowledge compared to counterparts without any inherited disorder (48.8%). Moreover, the family medical history significantly influenced knowledge levels (p-value < 0.001). Those with a family history of diseases showcased better knowledge (58%) in contrast to individuals without such histories (32.5%). While a difference in knowledge levels was observed between Saudi and non-Saudi individuals, it lacked statistical significance due to limited non-Saudi data (p-value = 0.106). On the same note, medication use did not significantly impact knowledge levels (p-value = 0.375). These findings highlight substantial disparities in knowledge levels among certain demographic groups concerning G6PD deficiency, particularly regarding gender, inherited disorders, and family medical history. Conversely, factors such as education, income, and nationality did not exhibit significant differences in knowledge levels.

**Table 4:** Association between knowledge and sociodemographic characteristics (n = 459)

-	-	Knowledge		P-values
		Good	Poor	
Sex	Male	67 (44.7%)	83 (55.3%)	0.008
	Female	179 (57.9%)	130 (42.1%)	
Age	20 - 40	98 (56%)	77 (44%)	0.42
	>40	148 (52.1%)	136 (47.9%)	
Marital Status	Married	243 (53.8%)	209 (46.2%)	0.56
	Divorced	3 (42.9%)	4 (57.1%)	
Education	Pre-secondary	8 (34.8%)	15 (65.2%)	0.174
	high school	41 (49.4%)	42 (50.6%)	
	Bachelor's/Diploma	178 (55.3%)	144 (44.7%)	
	Masters or Ph.D. graduate	19 (61.3%)	12 (38.7%)	
Family monthly income	< 5000	22 (50%)	22 (50%)	0.788
	5000 - 10000	55 (53.9%)	47 (46.1%)	
	10000 - 15000	73 (57%)	55 (43%)	
	> 15000	96 (51.9%)	89 (48.1%)	
Do you have any inherited disorders?	Yes	121 (59.6%)	82 (40.4%)	0.021
	No	125 (48.8%)	131 (51.2%)	
Family history of diseases	Yes	220 (58%)	159 (42%)	<0.001
	No	26 (32.5%)	54 (67.5%)	
Nationality	Saudi	243 (53.3%)	213 (46.7%)	0.106
	Non-Saudi	3 (100%)	0 (0%)	
Are you on any medication?	Yes	57 (50%)	57 (50%)	0.375
	No	189 (54.8%)	156 (45.2%)	

Logistic regression analysis, as seen in Table 5, was performed with significant demographic factors, including gender, individuals who have any inherited disorder, and family history of diseases for predicting the level of knowledge of G6PD deficiency anemia. Females were significantly associated with good knowledge of G6PD (OR=1.59, 95% CL=1.06-2.39; p=0.026), which indicates that the females who had good knowledge were 1.59 times more than males. Participants with a family history of diseases had good knowledge 2.52 times more than participants with no family history of chronic diseases (P < 0.001). Having an Inherited disorder was not a predictor of good knowledge.

**Table 5:** Multivariate analysis for predicting the level of knowledge (Good) about G6PD (n = 459) G6PD: glucose-6-phosphate dehydrogenase

-	Odds Ratios	95% C.I. for OR		P-values
		Lower	Upper	
Sex (Female)	1.59	1.06	2.39	0.026
Do you have any inherited disorders?	1.33	0.91	1.96	0.147
Family history of diseases	2.52	1.48	4.28	<0.001

## Discussion

G6PD, a disorder more prevalent among males due to its X-linked nature, affects more than 400 million individuals, making it the most common enzyme deficiency globally [1]. In Saudi Arabia, despite the apparent widespread presence of the disease, limited studies have investigated the awareness of the population regarding G6PD. Our study specifically targeted parents’ general knowledge of G6PD, revealing that 246 participants (53.6%) possessed a good understanding of the disease. This result was consistent with the findings of a study in Jazan by Hamali HA et al [8], wherein 50.5% of participants exhibited a good level of knowledge. Conversely, a study done by Alqahatni et al. [12] in Saudi Arabia reported poor knowledge among Saudi mothers, with only 39.4% demonstrating good knowledge. The favorable level of knowledge in our study is theorized to be the byproduct of either personal experience with the disease or a family history of chronic diseases.

When it came to the nomenclature of the disease, “G6PD deficiency anemia” was poorly recognized, with 363 (73.2%) participants having never heard of this name. However, the majority of our participants, specifically 377 (82.1%) participants, were familiar with its alternative name, “fava beans anemia”. These findings are in agreement with studies in Saudi Arabia conducted by Alqahatni et al. [12] and Jazan by Hamali HA et al. [8], as well as a study in Egypt by Kasemy ZA et al. [13], in which 95.9% of mothers had not heard the term G6PD deficiency. On the other hand, an Iraqi study reported that 75% of mothers had heard of G6PD deficiency [14]. This difference in nomenclature is thought to be influenced by the varying geographical locations and the social familiarity in each community.

Our study found that female participants had better knowledge of G6PD deficiency anemia than their male counterparts, which is in line with the Jazan study. This result is likely explained by the higher number of female participants in our study. Regarding inheritance, 369 participants (80.4%) believed the disease was inherited. However, 356 participants (77.5%) answered either "no" or "do not know" when asked whether the disease is linked more to a specific gender. Similar conclusions were reached in studies in Jazan [8], Iraq [14], and Bahrain [15], indicating a lack of knowledge regarding the mode of inheritance. The misconceptions are hypothesized to result from insufficient patient education and genetic counseling.

The relationship between medications and G6PD deficiency anemia attacks appears to be an area of major knowledge deficit. 234 (50.9%) participants did not know if medications were a possible trigger for an attack, and 48 (10.5%) participants believed medications could not induce a hemolytic attack at all. Studies in Iraq [14], Egypt [13], and Bahrain [15] shared the same results, reporting considerable percentages of participants who do not know the role of medications in triggering a G6PD attack (67%, 52%, and 37.2%, respectively). The main hypothesis credits this major misconception to improper patient education on medication side-effect profiles and a lack of public health campaigns. Participants’ primary source of information was either through search engines or their relatives.

Even though many participants demonstrated a fair understanding of G6PD symptoms, there was a substantial number of individuals who lacked awareness, posing a potential risk of heightened mortality and morbidity to their children if they were to have the disease. Therefore, it is crucial to prioritize parental education and initiate measures to enhance awareness within the general population. We recommend that primary healthcare physicians engage in proper patient education, provide suitable source material, and sincerely advocate for awareness campaigns about G6PD deficiency anemia to the general public.

Study strengths

The study pinpoints areas of misconceptions regarding G6PD deficiency, which allows guided practical interventions to alleviate these deficits. Moreover, the data collection and subsequent results and conclusions were completely cost-efficient. Lastly, a cross-sectional study design utilizing an online-based questionnaire to investigate the awareness of G6PD among parents enables the collection of data from a relatively large sample in a relatively short time using convenient and approachable methods, such as social media platforms, to reach the target population.

Study limitations

The nature of using an online survey could possibly skew the participants away from honest answers or ones that may be deemed unfavorable by the respondents. The difficulty to randomize the study is another important limitation when it comes to studies using online-based questionnaires. Additionally, the measurement of the level of knowledge based on the overall mean score of the participants is one of our study limitations, which resulted from the use of the Jazan study questionnaire, where similar prevalence trends of G6PD and hematological diseases are found. Furthermore, a small sample size makes it difficult to draw generalizable conclusions regarding the disease in other populations, especially when the location of the study and its endemic nature are taken into consideration.

## Conclusions

Our study highlighted significant widespread misinformation among the surveyed parents with an emphasis on the possible triggers, inheritance patterns, and symptoms and severity of G6PD. The need for initiatives to correct these misconceptions held by the general population has been made abundantly clear. Public health campaigns, reputable medical sources fit for patients, and the social media presence of trusted physicians are crucial in spreading awareness about G6PD deficiency by utilizing their reach and influence within the community.

## Appendices

Application

Identifying the level of awareness of a disease in the community aids in the application of awareness-raising measures that may decrease the morbidity and mortality of that disease.

Awareness of G6PD in the Eastern Province population

Section A: General questions

Date:      /      /

Sex: Female (        )    Male (          )   Age (          )               

Weight (Kg): ………Height (cm)………………City: ….........................................

  Telephone: (Optional…..........................................)             Date of birth:          /            /  

1.     What is your education level?

 Below high school

 Bachelor/Diploma

 Graduate (Master’s/PhD)

2.     Family income (SAR):

 <5000

 5000-10,000

 10,000-15,000 

 >15,000

3.     Nationality (check only one):

 Saudi                              Other ………………………………

4.     Marital status (check only one):

 Single        Married        Married and pregnant        Divorced with children

5.     Do you have any inherited disorder(s):  Yes    No , if yes which condition:

 Anemia                                Sickle cell anemia

 Thalassemia                        Other disease: …………………….

 I do not have any inherited disorder(s)

6.     Are you on any medication(s):  Yes    No 

If yes, write the name(s) of the medication(s): …………………….

7.     Do you have any relatives with inherited or chronic disorders?

 Yes           No     If yes, write the name(s) of the disorder(s): ……………………. 

8.     How many children do you have:

 1       2       3       4       More than 4       I do not have any children

9.     Do you have a child with G6PD deficiency:

 Yes           No           I do not know

Section B: Awareness of G6PD

10.  Have you ever heard of G6PD deficiency?

 Yes           No         

11.  Have you ever heard of Fava Bean anemia?

 Yes           No         

12.  Is G6PD deficiency a type of anemia?

 Yes           No           I do not know

13.  Do you believe that for a child to be affected by G6PD deficiency anemia, both parents must be carriers?

 Yes           No           I do not know

14.  Is G6PD deficiency an inherited disorder?

 Yes           No           I do not know

15.  Do you know if you have G6PD deficiency anemia?

 Yes           No           I do not know

16.  Do you have a family history of G6PD deficiency anemia?

 Yes           No           I do not know

17.  Is the gender of the child linked to G6PD deficiency anemia?

 Yes           No           I do not know

18.  Do you believe that the neonatal diagnosis of G6PD deficiency anemia is beneficial to your child’s care?

 Yes           No           I do not know

19.  Can Fava beans trigger an attack of G6PD deficiency anemia?

 Yes           No           I do not know

20.  Can certain medications trigger an attack of G6PD deficiency anemia?

 Yes           No           I do not know

21.  Are nausea, vomiting, anorexia, and diarrhea symptoms of a G6PD deficiency anemia attack?

 Yes           No           I do not know

22.  Is pallor one of the symptoms of a G6PD deficiency anemia attack?

 Yes           No           I do not know

23.  Is giddiness one of the symptoms of a G6PD deficiency anemia attack?

 Yes           No           I do not know

24.  Is jaundice one of the symptoms of a G6PD deficiency anemia attack?

 Yes           No           I do not know

25.  Is shortness of breath one of the symptoms of a G6PD deficiency anemia attack?

 Yes           No           I do not know

26.  Aside from the effects of G6PD deficiency anemia, have you noticed that it provides protection against the malaria parasite?

 Yes           No           I do not know

27.  Can splenectomy improve the health of patients with G6PD deficiency anemia? 

 Yes           No           I do not know

28.  Is laboratory testing for G6PD deficiency anemia included in premarital testing?

 Yes           No           I do not know

29.  Do you believe that G6PD deficiency anemia can lead to death or disability?

 Yes           No           I do not know

30.  Do you use any specific educational sources to increase your awareness regarding G6PD deficiency anemia?

 Yes           No           I do not know
